# Managerial coaches’ enacted behaviors and the beliefs that guide them: perspectives from managers and their coachees

**DOI:** 10.3389/fpsyg.2023.1154593

**Published:** 2024-01-05

**Authors:** Beth Adele, Andrea D. Ellinger

**Affiliations:** ^1^Department of Nonprofit Leadership, Petree College of Arts & Sciences, Oklahoma City University, Oklahoma, OK, United States; ^2^Department of Human Resource Development, Soules College of Business, The University of Texas at Tyler, Tyler, TX, United States

**Keywords:** managerial coaching, qualitative, coaching beliefs, manager as coach, managerial beliefs, coaching behaviors, employee coaching, critical incident technique

## Abstract

**Introduction:**

Managerial coaching is considered a powerful developmental intervention in the workplace that has gained tremendous popularity in recent years. A growing base of scholarship examining the efficacy of this form of coaching has identified numerous benefits, primarily for employees receiving this form of coaching, and some limited attention has focused on benefits for managers who coach. However, an important topic related to managerial coaching that has gone under-explored is the beliefs that managers have about coaching. Since beliefs often guide behaviors, obtaining a more robust understanding of the beliefs that guide managers who serve as coaches is warranted and several scholars have called for more research on this aspect of managerial coaching. Therefore, the study reported here presents a subset of findings that relate to the coaching behaviors that managers enact along with a comprehensive understanding of their guiding beliefs.

**Methods:**

These specific findings are drawn from a larger qualitative multi-case study employing an adaptation of the critical incident technique that was designed to examine the beliefs, behaviors, and learning and development outcomes for both managers who coach and their respective coachees. This larger study obtained perspectives about these aspects of managerial coaching from both the coaches and coachees which also reflects an approach seldom taken when researching managerial coaching.

**Results and Discussion:**

Four research questions are addressed here: 1) What are the behaviors enacted by managers who coach (facilitate the learning of) their employees from the perspective of managers; 2), what are the behaviors enacted by managers who coach (facilitate the learning of) their employees from the perspective of employees; 3) What are the beliefs held by managers who coach (facilitate the learning of) their employees from the perspective of managers; and, 4) What are the beliefs held by managers who coach (facilitate the learning of) their employees from the perspective of their employees (coachees)? In addition to thick rich descriptions that illustrate these findings, implications for theory, research, and practice are also discussed.

## 1. Introduction

Coaching has emerged as a powerful developmental approach in the workplace. There are various types of coaching and coaches; however, one form of coaching that is becoming more prominent is coaching by managers. Often referred to as managerial coaching, employee coaching, or the manager as coach, managerial coaching has been defined as a “supervisor or manager serving as a coach, or facilitator of learning, in which he or she [they] enacts specific behaviors that enable his/her [their] employee (coachee) to learn, develop, and improve his/her [their] performance” (Ellinger et al., 2011, p. 76). A growing base of research examining the efficacy of this type of coaching for employees suggests that employees who are coached receive many positive benefits that include learning, development, and enhanced performance (Hagen, 2012; Lawrence, 2017; Ellinger et al., 2018). Much less is known about the benefits that managers may derive from coaching, and another important void in the existing literature pertains to managers’ beliefs about coaching – in essence, why managers coach (Ellinger et al., 2018; Carvalho et al., 2022; Adele et al., 2023). Beliefs often represent assumptions that managers make, and terms like mental models, worldviews, and mindsets are often used interchangeably. Understanding beliefs is important because beliefs often guide behavior (Ellinger and Bostrom, 2002). At present, very little research has examined managers’ beliefs when serving as coaches or employee developers (Ellinger and Bostrom, 2002; Heslin et al., 2006; Misiukonis, 2011; Warhurst, 2013; Campbell and Evans, 2016). Thus, scholars contend that research is under-developed on managerial coaching beliefs and calls have been made for more research on this aspect of managerial coaching (Ellinger et al., 2018; Carvalho et al., 2022).

Therefore, the purpose of the study reported here is to examine the beliefs and behaviors of managers who coach, both from their perspective and that of their coachees. More specifically, based upon these enacted behaviors, the primary researcher sought to explore managers’ beliefs about coaching from the perspectives of managers and their coachees. These findings represent a subset of findings drawn from a larger study. In the sections that follow, the relevant extant literature on managerial coaching, behaviors, and beliefs is reviewed. The design and methods undertaken to conduct this research is described. The specific findings are presented and illustrated and discussed and situated within the existing literature. Implications for theory, research, and practice are considered, and the article concludes with limitations and directions for future research.

### 1.1. Review of the literature and conceptual grounding

This section reviews the extant literature about managerial coaching, managerial coaching behaviors, and the limited research that has explored managerial coaching beliefs. It then introduces concepts drawn from Argyris and Schön (1996) that provide insight about managerial reasoning and beliefs which serves as the theoretical underpinning for this qualitative multi-case study.

#### 1.1.1. Managerial coaching

Coaching can be traced back in history; however, its application in the management literature was popularized by Orth et al. (1987) and Evered and Selman (1989). In particular, Evered and Selman (1989) considered coaching to be a core managerial activity. Various managerial taxonomies have suggested that managers have training, coaching, and development roles, and some early research explored sales managers’ coaching behaviors (Ellinger et al., 2018). However, the lack of an in-depth understanding of the precise coaching behaviors that managers enact resulted in calls for research that have been addressed by a number of scholars (Ellinger, 1997; Ellinger et al., 1999; Beattie, 2002; McLean et al., 2005; Park et al., 2008; Dixey, 2015). In subsequent comparative analyses of studies examining managerial coaching behavior and behaviors associated with managerial and leadership effectiveness, Hamlin et al. (2006) conceived managerial coaching to be at the heart of managerial effectiveness. Since then, managerial coaching research has flourished with studies examining its efficacy, outcomes for coachees, with limited attention being given to outcomes for managers and explications of managers’ beliefs about coaching (Ellinger and Bostrom, 2002; Hunt and Weintraub, 2002; Misiukonis, 2011; Campbell and Evans, 2016). The next sections offer a brief synthesis of research on managerial coaching behaviors and beliefs.

##### 1.1.1.1. Behaviors of managerial coaches

An established base of literature identifying managerial coaching behaviors and the requisite skills managers need to coach exists. Beyond behaviors articulated by Graham et al. (1994) and Orth et al. (1987), Ellinger (1997) examined exemplary managers who facilitate the learning of their employees, or as they referred to, coach them. Ellinger (1997) and Ellinger et al. (1999) developed a taxonomy that identified 13 managers’ coaching behaviors within 2 clusters, those that facilitated and those that empowered employees (coachees). Following this research, Beattie (2002) conducted a similar qualitative study in the Scottish social service context which resulted in 22 discrete effective facilitative behaviors that were classified into 1 of 9 categories. Shortly thereafter, Hamlin et al. (2006) conducted a cross-cultural comparative analysis of the managerial coaching behaviors identified by Ellinger (1997), Ellinger and et al. (1999), and Beattie (2002) along with Hamlin’s (2004) managerial and leadership effectiveness behaviors and found a high degree of congruency among these behaviors. Additional support for these behaviors was reported in a subsequent literature review of 15 studies by Lawrence (2017) and other scholars’ research (Beattie, 2002; Amy, 2008; Hagen and Peterson, 2014) which appear in Table 1. Although Table 1 includes content that reflects behavioral and skill-based perspectives, we adopt a behavioral perspective in the findings we report here.

**Table 1:** Managerial coaching behaviors and skills / dimensions of coaching scales.

Ellinger (1997) Ellinger et al. (1999)	Beattie (2002)	Ellinger et al. (2003) ^*^	Heslin et al. (2006) ^*^	Amy (2008)	Park et al. (2008) ^*^	Gilley et al. (2010) ^*^	Gregory and Levy (2010) ^*^	David and Matu (2013) ^**^
Behavior clusters	Behavior clusters	Coaching behavior measure	Behavioral observation Scale	Behavior clusters	Measurement model of coaching skills	Skills and behaviors of managerial coaching	Predicted quality of the coaching relationship	Managerial coaching assessment System
Empowering Cluster of Behaviors: Question Framing to encourage employees to think through issues Being a resource – removing obstacles Transferring ownership to employees Holding back – not providing the answers Facilitating Cluster of Behaviors: Providing feedback to employees Soliciting feedback from employees Working it out together – talking it through Creating and promoting a learning environment Setting and communicating expectations Stepping into other to shift perspectives Broadening employees’ perspectives – getting them to see differently Using analogies, scenarios and examples Engaging others to facilitate learning	Facilitating Cluster of Behaviors: Thinking – reflective or prospective thinking Informing – sharing knowledge Empowering – delegation, trust Assessing – feedback and recognition, identifying developmental needs Advising – instruction, coaching, guidance, counselling Being professional – role model, standard setting, planning and preparation Caring – support, encouragement, approachable reassurance, commitment/ involvement, empathy Developing others Challenging employees to stretch themselves	Using analogies, scenarios, and examples Broadening employees’ perspectives – getting them to see things differently Providing feedback to employees Soliciting feedback from employees Being a resource – removing obstacles Question framing to encourage employees to think through issues Setting and communicating expectations Stepping into other to shift perspectives	Guidance Facilitation Inspiration	Facilitating: Asking questions of followers Clarifying to establish mutual understanding Delegating learning projects to followers Teaching through overt instruction and sharing information Upholding existing standards and maintain accountability Problem Solving and Decision Making: Advising through suggestions and constructive confrontation Consulting relevant stakeholders before making decisions Empowering followers to make autonomous decisions Experimenting through brainstorming and exploring alternatives Communicating and Relating: Customizing learning episodes to address individual needs Emoting to connect with followers Motivating by sharing recognition and providing incentives Perceiving followers’ needs by reading them accurately Developing: Advancing systems and technology Balancing individual and organizational concerns Cultivating subject matter experts Documenting best practices Documenting processes and procedures Involving upper management Ineffective Behaviors: Failing to respond to followers thereby neglecting learning Relating to followers in an authoritarian manner Responding to followers in a defensive manner	Open communication Team approach Value people Accept ambiguity Develop people	Possess skills necessary for the job Communication Motivation Encourage growth and development	Genuineness of the relationship Effective communication Comfort with the relationship Facilitating development	Encourage others to find own solutions Empower others Offer guidance rather than solutions Offer positive feedback Offer negative constructive feedback Ask for feedback Develop plans Offer learning opportunities Set expectations Establish clear goals Look at things from others’ perspective Encourage different perspective Use analogies, scenarios, examples Bring in others to facilitate learning when required

* (Hagen and Peterson (2014); Lawrence (2017), pp. 49-50).

** (Lawrence (2017), pp. 49-50).

##### 1.1.1.2. Beliefs of managerial coaches

Despite the prevalence of managerial coaching in organizational contexts, Carvalho et al. (2022) acknowledged that “more dialog and research that uncovers the motivations, challenges, and benefits managers accrue from coaching…seems warranted” (p. 286). Understanding the “why” aspect of why managers coach and most especially the beliefs managers may hold in the black box of their minds has been considerably under-researched (Ellinger and Bostrom, 2002; Misiukonis, 2011; Campbell and Evans, 2016). Terms such as beliefs, mental models and worldviews (Senge, 2006), mindsets (Hunt and Weintraub, 2017), and theory-in-use (Argyris, 1991) are often used interchangeably, but they are important to understand because they often influence “how we understand the world and how we take action” (Senge, 2006, p. 8). However, the action theories of managers who coach, or their beliefs or mindsets, have not been widely researched and according to Barrero (2022), “until recently, few studies examined manager beliefs empirically” (p. 640–641).

In terms of the limited research that has explored managers’ beliefs about coaching, Ellinger and Bostrom (2002) were among the first scholars to analyze exemplary managers’ coaching beliefs. They identified five clusters of beliefs that formed three major categories of beliefs as presented in Table 2. They also developed a conceptual framework suggesting that a manager’s beliefs, through the role identity of a manager as a coach or facilitator of learning, enact behaviors that elicit learner outcomes, manager outcomes, and organizational outcomes. Misiukonis (2011) was next to embark upon a phenomenological study to examine middle managers’ beliefs about organizational coaching and their coaching practices which resulted in seven clusters which formed four categories of beliefs also depicted in Table 2. Anderson’s (2013) subsequent research revealed that managers’ relationships with their employees and their occupational self-efficacy were predictive of their coaching behaviors. Campbell and Evans (2016) continued to explore beliefs of managers as facilitators of learning in a public utility organization using Ellinger and Bostrom’s (2002) model.

**Table 2:** Beliefs held by managers as facilitators of learning.

Ellinger and Bostrom (2002, p. 155–156, p. 164)	Campbell and Evans (2016, p. 80)	Misiukonis (2011, p. 57)
Category 1: Beliefs held by facilitators of learning about their roles and capabilities: Beliefs about roles: My role is to facilitate learning and development – this is what I do Coaching is all about people – helping them to grow and develop There is a difference between coaching and management – role distinction Management is telling people what to do Beliefs about capabilities: I care enough to help them I have skills and process capabilities that I can apply I have a reservoir of experiences that I can apply Establishing trust and building relationships is critical Category 2: Beliefs held by facilitators of learning about the learning process and learning: Beliefs about the learning process: Feedback is important Integrate learning with work Learners must be encouraged to learn for themselves Beliefs about learning: Learning is important Learning is ongoing Learning is shared Category 3: Beliefs about learners: Beliefs about learners: Learners are very capable Learning must be willing to learn – learning is not one-way Learners need a solid information foundation Learnings need to understand the ‘why’s	Beliefs held by managers about their roles, skills, and capabilities: Facilitator skills, experience, and self-belief Managing skills risk and succession planning Beliefs held by managers about learning and the learning process: Learning is social and experiential Learning delivers business results Learning builds confidence and self-belief Learning is enabled through feedback Beliefs held by managers about learners: People are individuals People need to take ownership and problem-solve People need help to see the bigger picture People need support to develop confidence Beliefs held by managers about leadership and the environment: Creating the right environment Leaders are role models	Beliefs about self-efficacy and own roles: Beliefs about self-efficacy and capabilities in coaching Beliefs about own role as a middle manager Beliefs about organisation and management: Beliefs about organization Beliefs about management Beliefs about subordinates: Beliefs about subordinates (coachees) Beliefs about coaching: Beliefs about coaching as an approach Beliefs about coaching as a practice in organisations

Many of Campbell and Evans’s (2016) categorized beliefs reinforced those previously identified by Ellinger and Bostrom (2002), however, they found two new beliefs that reflect managers’ beliefs about serving as role models, and the beliefs that managers should manage skill-related risk and succession planning also noted in Table 2. For Hunt and Weintraub (2002, 2017), the coaching mindset is a set of beliefs and attitudes that managers have who become effective coaches. Their coaching mindset consists of: the belief that having a good relationship with their reports is important, an attitude of helpfulness, less need for control, empathy in dealing with others, openness to personal learning and receiving feedback, the belief in setting high standards, a desire to help others to develop, employee development is not a ‘sink or swim’ approach, people are not to be “fixed,” and a belief that most people want to learn (Hunt and Weintraub, 2002, 2017). Despite these specific studies, calls for more research on managerial coaching beliefs have been made (Ellinger et al., 2018; Carvalho et al., 2022).

#### 1.1.2. Conceptual grounding

The study reported here drew upon Argyris and Schön’s (1996) and Argyris’ (1991) concept of theory-in-use to better understand managerial coaching behavior as reported by managers and their respective employees. Over the years, Argyris and Schön (1996), Schön (1983), and Argyris (1991) have engaged in research on managerial reasoning and action to better understand the disconnects between two types of theories of action, espoused theory of action, and theory-in-use. For these scholars, theory-in-use reflects an action theory of the actual behaviors that managers enact. They suggest that there is often a discrepancy between what they term, espoused theory of action, which is an action theory that reflects what managers think they are doing versus how they really act in practice. They contend that defensive reasoning plays a part in this inconsistency but that managers can be taught to identify such inconsistencies in their theories of actions so that they can reason productively and that it is possible to “change the master programs in their heads and thus reshape their behavior” (p. 106).

## 2. Materials and methods

This section reviews the design of the study, sample selection, data collection, and analysis approaches employed.

### 2.1. Design of the study

The subset of findings reported here are drawn from a larger qualitative, multi-case study that used an adaptation of the Critical Incident Technique (Ellinger and Watkins, 1998; Chell, 2004; Gremler, 2004; Butterfield et al., 2005, 2009; Watkins et al., 2022) and semi-structured interviews to collect the data. The primary author and researcher embraced the epistemological position of interpretivism and the ontological orientation of constructionism which suggest that people construct their own meaning of the world from their unique experiences (Privitera and Ahlgrim-Delzell, 2019). The intent of the primary author and researcher was to obtain an “empathetic understanding of human action rather than with the forces that are deemed to act on it” along with the beliefs that guide such human action (Bryman and Bell, 2011, p. 16). A total of 12 managers and one of each of their respective, directly-reporting employees comprised the managerial coaching dyads and each dyad served as one case, bounded system, reflecting the multi-case design (Merriam and Tisdell, 2016; McWhorter and Ellinger, 2018; Yin, 2018).

Four research questions are addressed using the subset of data from the larger study: 1. What behaviors are enacted by managers who coach (facilitate their employees’ learning) from the perspective of the managers?; 2. What behaviors are enacted by managers who coach (facilitate the learning of their employees) from the perspective of their employees (coachees)?; 3. What beliefs are held by managers who coach (facilitate the learning of their employees) from the perspective of managers?; and, 4. What beliefs are held by managers who coach (facilitate the learning of their employees) from the perspective of their employees (coachees)?

#### 2.1.1. Sample selection

The 12 managers were recruited through nominations from two third-party, coach/trainers, who were external professionals located in the south-central United States. The nominating professionals practiced in the networking and consulting industry allowing them to identify exemplary managers who coach (facilitate the learning of) their employees. The nominators were instructed to use the following criteria to identify and nominate exemplary managers and their respective subordinate employees: 1. the nominating professional must perceive the manager to be an exemplary managerial coach (facilitator of learning); 2. the manager must have had a coaching (learning facilitation) relationship with their employee for at least 1 year; 3. the manager must identify with serving as a coach/developmental manager/leader of their directly reporting employee in the workplace and recall developmental interactions with their employees; 4. the manager must be willing to nominate their employee (coachee) to participate in the study with the understanding that the nominated employee will be receptive to participating in the study; and, 5. the manager must be available for a face-to-face interview for up to 60 minutes and their employee must also be available to participate in a separate face-to-face interview for up to 60 minutes. After being contacted by the primary researcher, the nominated managers, upon written consent to participate in the study, were asked to nominate one of their directly-reporting employees for a separate, individual interview.

In addition to collecting consent forms from participants, some demographic and background information was collected that included title, time as a manager, self-reported gender, number of employees in the organization, organization industry, and geography of organization. The managers reported a range of 7 to 35 years of managerial experience with an average of 19 years. Table 3 presents a summary of the collected information for each participant and their assigned pseudonyms.

**Table 3:** Summary of sample demographics.

Participant set	Pseudonym	Self-reported gender	Title	NAICS 2-Number code industry	Firm size
1	Noel	F	CFO/COO	Health care and social assistance	100–249
1	Natalie	F	Human resources	Health care and social assistance	100–249
2	Tom	M	CEO/Owner	Professional, Scientific, and technical services	<20
2	Tammy	F	Designer/project manager	Professional, Scientific, and technical services	<20
3	Jim	M	CEO	Heating and air-conditioning	20–49
3	John	M	Electrical manager	Heating and air-conditioning	20–49
4	Matt	M	Owner/Manager	Manufacturing	20–49
4	Max	M	Production manager	Manufacturing	20–49
5	Scott	M	Engineering manager	Manufacturing	20–49
5	Simon	M	Manufacturing engineer	Manufacturing	20–49
6	Brooke	F	Vice President of sales and marketing	Construction	<20
6	Brenda	F	Sales assistant	Construction	<20
7	Ken	M	Director of sales and marketing	Finance and insurance	100–249
7	Kyle	M	Sales manager	Finance and insurance	100–249
8	Darla	F	Senior district manager	Retail trade	>1,000
8	Daisy	F	Executive sales rep	Retail trade	>1,000
9	Philip	M	Fellow	Professional, Scientific, and technical services	100–249
9	Penny	F	Software developer	Professional, Scientific, and technical services	100–249
10	Anna	F	President/CEO	Health care and social assistance	100–249
10	Allison	F	Executive director	Health care and social assistance	100–249
11	Charles	M	CEO/Co-Owner	Construction	<20
11	Carla	F	Vice president of sales and marketing	Construction	<20
12	Randy	M	President	Manufacturing	20–49
12	Roger	M	Marketing manager	Manufacturing	20–49

#### 2.1.2. Data collection and analysis

An adaptation of the Critical Incident Technique (CIT) (Ellinger and Watkins, 1998; Chell, 2004; Gremler, 2004; Butterfield et al., 2005, 2009; Watkins et al., 2022) and semi-structured interviews were the primary data collection approaches. Ellinger and Watkins (1998) adapted Flanagan’s (1954) CIT approach by incorporating elements of the Marsick and Watkins (1990) Informal and Incidental Learning model to capture context, reasoning, and meaning, and to acquire insights pertaining to effective and ineffective behaviors and outcomes in the larger study.

The primary researcher conducted in-person, semi-structured interviews seeking to obtain at least 2 critical incidents from each individual participant. Separate interviews lasting approximately 60 minutes were conducted with each participant. Twelve managers and their respective employees, 24 in total, were interviewed gleaning 48 or more critical incidents. All interviews were digitally recorded and transcribed with participants’ permissions, and field notes and observations were collected during the interviews to assist in the analysis phase.

Data analysis consisted of both deductive and inductive approaches. The initial formation of broad *a priori* content categories was guided by previous research and the research questions that were focused on managers and employees regarding their behaviors, beliefs, and outcomes in the larger study. This deductive approach using *a priori* content categories was an initial sorting device for the data collected from the critical incidents and the semi-structured interviews. These data were sorted into these initial broad categories using definitions drawn from literature regarding behaviors, beliefs, and outcomes. For the subset of findings that are reported in this study, behaviors were defined as actions, or set of actions that managers enact when they perceive they are coaching their employees (Ellinger et al., 1999). Beliefs were defined as “a set of personal and professional assumptions and worldviews that guide action” (Ellinger et al., 1999, p. 111).

Following the deductive approach using *a priori* content categories for the initial sorting using content analysis (Weber, 1990), the researchers employed inductive constant comparative analysis (Strauss and Corbin, 1990) within these broader content categories to derive themes and subthemes that emerged within the categories. Once the themes and subthemes were established, the complete transcriptions of the interviews were coded using computer-assisted qualitative data analysis software, NVivo, published by QSR International Pty Ltd.

To confirm trustworthiness and authenticity within the qualitative tradition, various strategies were used (Merriam and Tisdell, 2016). To achieve triangulation of data, multiple sources of data were obtained including the semi-structured interviews with managers and their respective employees, critical incidents from each member of each dyad, observations, and field notes. Member checks were conducted initially by returning the entire, verbatim interview transcriptions to each respective participant giving them the opportunity to clarify or add to their responses prior to data analysis. To support the dependability and confirmability of the study, dissertation committee members reviewed transcripts and contributed to the inductive constant comparative analysis through peer reviews. Respondent validation was performed by sending the derived themes and subthemes to the participants and the nominating professionals to review for plausibility. Detailed, rich descriptions of participants’ reporting of critical incidents and the thematic analysis were developed, and an audit trail was implemented and managed throughout the duration of the study. Digital and hard copy storage of transcripts and all verbal and written communication were kept as components of the audit trail to enhance the rigor of the study.

## 3. Findings

This section presents this study’s major findings addressing the four aforementioned research questions. Tables 4 and 5 each provide a summary of these themes and subthemes which are illustrated with subsequent participant quotations.

**Table 4:** Managers’ behaviors themes when managers are serving as coaches.

Managers’ perspectives of their enacted behaviors when serving as coaches	Employees’ perspectives as coachees of managers’ enacted behaviors when serving as coaches
Managers’ behaviors	
*Manages employees in role as developmental manager – 11/35* Providing feedback – 6/8 Prioritizing and organizing – 5/10 Assessing employee behavior – 4/8 Providing accountability – 2/5 Providing resources – 1/2 Delegating – 1/1 Accepting the managerial role – 1/1 *Fosters professional learning environment – 11/27* Asking employee to self-reflect – 7/12 Intentionally scheduling meetings – 6/8 Listening – 4/4 Leading by example – 3/3 *Empowers and develops employees – 10/35* Empowering others – 8/21 Promoting and developing employees – 5/12 Teaching technical skill – 2/2 *Fosters open, relational communication – 7/21* Establishing rapport through trust and communication – 7/19 Adjusting style for individual employees – 1/1 Observing employees managing – 1/1	*Empowers and develops employees – 12/48* Developing employees – 10/22 Including others in problem solving – 5/11 Exhibiting patience-forgiveness – 5/6 Trusting employees – 3/3 Supporting employees – 2/4 Participating in business – 2/2 *Fosters open, relational communication – 11/44* Accepting-soliciting feedback −10/21 Communicating expectations – 7/11 Listening – 5/8 Communicating managerial beliefs – 2/2 Encouraging questions – 1/1 *Fosters professional learning environment – 11/21* Intentionally scheduling meetings – 5/6 Leading by example – 4/8 Teaching verbally – 3/4 Being accessible – 1/3 *Managing employees in role as developmental manager – 9/20* Providing feedback-correction – 7/14 Prioritizing – 3/4 Providing accountability – 2/2

Managers’ perspectives of their enacted behaviors when serving as coaches	Employees’ perspectives as coachees of managers’ enacted behaviors when serving as coaches
Managers’ capacity-building behavior	
*Commitment to self-learning – 11/21*	*Models self-induced learning – 4/4*

Numbers provided in format X/Y – X represents the number of participants (out of 12), and Y represents the number of references.

**Table 5:** Managers’ beliefs themes when managers are serving as coaches.

Managers’ beliefs	
Managers’ perspectives of their beliefs when serving as coaches	Employees’ perspectives as coachees of managers’ beliefs when Serving as coaches
*Beliefs about self-awareness – 11/31* Belief about seeking learning opportunities – 6/9 Belief that one must be self-aware – 5/10 Belief about own personal strengths and weaknesses – 5/6 Belief about learning from employees – 2/4 Belief that one can only change oneself – 1/2 *Beliefs about learning – 11/26* Belief that learning continually happens – 6/7 Belief that all should and can learn – 5/11 Belief that uncomfortable experiences lead to learning – 2/4 Belief that one must seek to understand in order to learn – 2/2 Belief that learning is fun – 1/1 *Beliefs about my role as manager – 9/23* Belief that managers should develop employees – 6/7 Belief that the manager role is employee-role alignment 6/6 Belief that manager role is employee learning and success – 3/7 Belief that managers are to hold employees accountable – 2/2 Belief that managing comes naturally – 1/1 *Beliefs about context for facilitating learning – 8/20* Belief that trust and honesty are important – 4/10 Belief that psychological safety is important – 3/4 Belief to be intentional about one-on-ones – 3/3 Belief in a culture of respect – 1/1 Belief in systems – 1/1 *Beliefs about knowing my employees – 8/18* Belief that individuals are different – 4/9 Belief about employee strengths – 4/4 Belief about relying on employee skills – 3/3 Belief that people do not want to disappoint – 1/1 Belief that employee body language is telling – 1/1 *Beliefs about how to manage more developmentally – 8/16* Belief that authoritative management is bad – 3/4 Belief about managing with flexibility – 3/3 Belief that you lead by example – 2/5 Belief that communication is important – 1/1 Belief that one should keep a big-picture perspective – 1/1 Belief that listening is important – 1/1 Belief that managing is like parenting – 1/1 *Beliefs about knowing each other – 5/10* Belief that informal relationships are important – 2/4	*Beliefs about employee – 5/6* Belief in employee capabilities – 3/4 Belief about trusting the employee – 2/3 *Beliefs about management style – 4/5* Belief that the manager values relationships – 2/2 Belief that the manager has positive intent – 1/1 Belief that the manager values communication – 1/1 *Belief about learning – 2/2*

Numbers provided in format X/Y – X represents the number of participants (out of 12), and Y represents the number of references.

### 3.1. Behaviors of managerial coaches

Five themes with 17 subthemes emerged when analyzing the data that were categorized as *Managers’ Perspectives of Their Enacted Behaviors When Serving as Coaches*. The broad themes include *manages employees in roles as developmental manager*, *fosters professional learning environment*, *empowers and develops employees*, *fosters open, relational communication*, and one that will be presented later in this section, *commitment to self-learning*. The themes and subthemes are listed in Table 4 in order of frequency.

Eleven of the 12 managers spoke of their *management of employees as a part of their developmental managerial roles* resulting in 35 references about the behavior. The behaviors enacted pertain to what the managers perceive as the duties that fall within the title of manager. Seven subthemes were identified included *providing feedback*, *prioritizing and organizing*, *assessing employee behavior*, *providing accountability*, *providing resources*, *delegating*, and *accepting the managerial role*. This theme is depicted through the following sample of quotes:

- Randy spoke of providing feedback with a guided approach in “helping her [employee] put a little more structure to it, maybe changing the direction a little bit, or focusing it, and refining it, and then handing it back to her.”
- Noel expressed assessing her employees, “My entire career has been spent in some degree assessing human behavior…I pay a lot of attention to body language and what it’s saying because it’s telling me a story that’s so much greater than the words that are going to come out of someone’s mouth.”
- Matt spoke of taking an active role in watching: “I noticed his hours…I do walk-throughs…I’m an observer…I just do more observations.”
- Randy summarized managers who provided accountability. He said, “I am going to hold her accountable for things…My overarching objective is to keep her focused on the things that…um…behaviors and habits and projects that she needs to focus on to be successful.”
- Randy alluded to providing resources stating, “And at the same time providing the resources, which is what I do for anybody that reports to me, providing the resources that she needs to get the job done…So, what I do is help [employee] break the goals down into the discreet steps, and identify the resources she might not have yet or what she does have or does not have, and then we figure out together ok are we going to get you the resources.”

Eleven of the 12 managers spoke of their *fostering a professional learning environment* resulting in 27 references about the behavior. Four subthemes were identified including *fostering a learning environment*, *asking the employee to self-reflect*, *intentionally scheduling meetings*, and *leading by example*. This theme is illustrated through the following sample of quotes:

- Charles fosters his employee’s learning by asking the employee to self-reflect by “helping her think through her strategies and tactics, and basically just questioning her, in a constructive way, on her thought processes and decision processes, and making sure that she is thinking through the logic…and not either jumping to conclusions or going down a rabbit hole…”
- Brooke mentioned holding meetings and what happens in those meetings as well to aid in her employee’s learning. She said, “Every week, we sit down on what we call a one-on-one…I just simply asked her, ‘I have a feeling this is how you learn, am I right? Do you agree with this?’”
- Philip described not only a formal meeting process, but an informal one. He said, “We have a scheduled one-on-one that’s on the calendar for an hour once a week and when that does not happen, we’ll schedule it another time or at the very least, touch base at another time during the week…if she runs into road blocks in between, she’ll call me and if I need an update or get curious or have some random idea, I’ll call her.”
- Noel depicts the behavior of listening saying “I spend a lot of time, when I say listening, I’m listening for what their words are telling me, but more importantly, listening to what their body is telling me.”

Ten of the 12 managers spoke of *empowering and developing employees* resulting in 35 references about the behavior. Three subthemes were identified including *empowering others*, *promoting and developing employees*, and *teaching technical skill*. This theme is demonstrated through the following sample of quotes:

- Tom spoke of promoting and developing their employee saying “…we also talk about what are your long term career goals, because we do not expect everyone to work here forever…so if there, especially if there are training, educational, self-improvement opportunities we can help with that have an overlap…in this skill set and will help at [organization name] and will help you in your future position, even if it’s kind of custom for them or specific to them and the training would not make any sense or as much sense for anyone else, I still encourage those types of opportunities.”
- Matt prepared his employee for his career development and said, “I want you to start paying attention to this environment as a whole, not just the production part that you are overseeing” because “more than likely, he is going to be the guy I move into overseeing, maybe, the whole operation.”
- Scott spoke of teaching his employee technical skills mentioning, “I was showing him how to, you know, put some of that down, as far as making formulas and Excel spreadsheets and stuff…probably showing him how to do some of that and make some of his life easier, you know.”

Seven of the 12 managers spoke of *fostering open, relational communication* resulting in 21 references about the behavior. Three subthemes were identified including *establishing rapport through trust and communication*, *adjusting style for individual employees*, and *observing employees managing*. This theme is depicted through the following sample of quotes:

- Charles admitted establishing rapport saying, “Quite frankly a lot of times, I do not know what to do either, let us just talk it out, let us figure it out, and maybe between the two of us [manager and employee], we will come up with something.”
- Charles also illustrated adjusting style by saying, “The approach is similar, but it is adjusted for all my reports because they all communicate differently, and they all receive information differently.”

Five themes with 18 subthemes emerged from the *Employees’ Perspectives as Coachees of Managers’ Enacted Behaviors as Coaches*. The broad themes include *empowers and develops employees*, *fosters open, relational communication*; *fosters professional learning environment*; *managing employees in role as developmental manager*; and one that will be presented later in this section, *models self-induced learning*. The themes and subthemes are listed in Table 4 in order of frequency.

Twelve of the 12 employees spoke of their managers’ behaviors of *empowering and developing employees* resulting in 48 references about the behavior. Six subthemes were identified including *developing employees, including others in problem solving*, *exhibiting patience-forgiveness*, *trusting employees*, *supporting employees*, and *participating in business*. This theme is illustrated through the following sample of quotes:

- Kyle acknowledged, “He [manager] was in touch with every person and always would be promoting other people and trying to get them to be better.”
- Kyle opined, “I think she pushes me to be at my full potential, where she constantly says I’m still not there yet, like she thinks I can do something…I know there are other things she thinks I can do.”
- Simon spoke of his manager including others in problem solving stating, “But what [manager] gave me was to try to get other people to talk about why they thought it wasn’t working right. Helping people get on board with the fix rather than just stand around griping about how it should run better.”
- Daisy demonstrated her manager exhibiting patience-forgiveness stating, “[Manager] is now like, send us your success stories, but send your failures in too because we can learn more about what we are doing really bad than we can knowing what we are doing really well.”
- Penny said, “[Manager] is very patient and understanding.”
- Carla spoke of her manager trusting her by mentioning, “It took over a year learning to trust each other in order for that level of communication to happen”
- Daisy stated, “[Manager] does not try to change me or my behavior to be something that I’m not.”
- Penny stated that her manager supports her saying, “[Manager] has always ended a conversation with, ‘Is there anything else I can do for you…How can I help you? Do you need anything from me?’”
- Natalie and Daisy illustrated the subtheme of managers participating in business saying, “[Manager] was over there for the week. She stood in the…meetings; she stood in speaking to family members; she stood in; everything aside from actually doing [the employees’ jobs],” and “As a manager, she rides with us.”

Eleven of the 12 employees spoke of their managers *fostering open, relational communication resulting* in 44 references about the behavior. Five subthemes were identified including *accepting-soliciting feedback*, *communicating expectations*, *listening*, *communicating managerial beliefs*, and *encouraging questions*. This theme is illustrated through the following sample of quotes:

- Tammy pronounced, “He’s responsive to feedback and wants to improve and those are all good things.”
- Max said, “He appreciated that I gave him the feedback…he wants the feedback.”
- Carla mentioned, “[Manager] is the owner and CEO; and, if you are in a meeting with me, you would think I was part owner because of the way he treats me. Everyone in our company is allowed to have a voice and an opinion and communicate.”
- Natalie said her manager encourages questions and communicates her managerial beliefs stating, “She did not make me feel dumb…she calls all of us an investment.”
- Allison stated, “She [manager] listens. She makes sure she understands what someone else is feeling…she’s a real feeler.”

Eleven of the 12 employees spoke of their managers *fostering professional learning environments* resulting in 21 references about the behavior. Four subthemes were identified including *intentionally scheduling meetings*, *leading by example*, *teaching verbally*, and *being accessible*. This theme is demonstrated through the following sample of quotes:

- Allison described her manager executing a more unstructured method of fostering a professional learning environment stating, “[Manager’s] style of teaching is to model. She models the behavior that she expects or that she believes to be a best practice.”
- Simon spoke of his manager teaching verbally saying, “[Manager] tells me about stuff he’s done I the past…But just him telling me about some of the stuff that went on at [former company]…and listen to him talk about how he worked with people to get around a problem…a lot of different things he’s just told me as we sit around talking sticks in my head as methods to get around a problem.”
- Roger described his manager being accessible expressing, “[Manager’s] so accessible, and because I feel confident and comfortable talking with him on virtually any subject that has to do with the company, it certainly lends a level of comfort to my daily life.”

Nine of the 12 employees spoke of their managers’ *developmental management of employees* as a part of their managerial roles resulting in 20 references about the behavior. Three subthemes were identified including *providing feedback-correction*, *prioritizing*, and *providing accountability*. This theme is illustrated through the following sample of quotes:

- Kyle remarked about his manager providing feedback-correction, “[Manager’s] given me advice on improving my communication, executive communication, board, different layers of communication you have.”
- Tammy spoke of her manager prioritizing stating, “[Manager] helps me reign it in, he helps me figure out my priority list and my schedule and helps me wrap my head around it.”
- Natalie alluding to her manager providing accountability saying, “That’s a big thing she always tells people… ‘You’re telling yourself a story. Where did you base that off of? Are you telling yourself a story?’”

#### 3.1.1. Managers’ commitment to learning

Eleven of the 12 managers also spoke of their *commitment to self-learning*, a behavior that does not necessarily directly facilitate the learning of their employees, but rather builds the managers’ capacity to facilitate learning. Thus, it is a behavior enacted by the manager which indirectly facilitates the learning of his/her employee.

- Matt admitted, “…anytime I learn something from one of my subordinates, I take it as a…I log it to myself as a resource. In other words, if I learn from them in one area now, they are a resource for me.”
- Ken stated, “…alright, we got into this situation where one of the things that I felt personally was a real, kind of, lever moving my own development forward was getting a greater understanding of my own derailers, and things that could have had an impact on my own performance; and as I learned more about that, and I’ve had a lot of opportunity to have some good coaching from some executive coaches, it’s allowed me to become a better manager by maybe, de-railing less, being a better communicator, working better with others…”

The employees also reflected on their managers’ commitment to learning. Four of the 12 employees spoke of their managers *modeling self-induced learning* resulting in 4 references about the behavior. This behavior was separated into a different theme from the managers’ perspectives because of the indirect relationship the managers’ enactment of self-learning has on employees’ learning. However, when perceived by the employee, managers’ self-induced learning presents itself as modeling behavior.

- Simon remembered how his manager engaged in self-learning when he was new to his management positions. He disclosed, “When he first got here, I think his first 6 months or so going around the whole company, watching everything that was going on, trying to figure out what do we do, how do we do it.”
- Kyle shared, “…how does it impact us…evidence of learning…I am a younger age than him, and get to realize the same thing…so if I catch myself doing something similar, I remember those times and remember how he’s worked hard to change and I know he has, because he’s told me he’s had to work hard to change, even with his relationship at home and how he values work versus home life, and I’m trying to do the same thing…”

### 3.2. Beliefs of managerial coaches

Seven themes with 38 subthemes emerged when analyzing the data that were categorized as *Managers’ Perspectives of Their Beliefs When Serving as Coaches*. The broad themes include *beliefs about self-awareness*, *beliefs about learning*, *beliefs about my role as manager*, *beliefs about context for facilitating learning*, *beliefs about knowing my employees*, *beliefs about how to manage more developmentally*, and *beliefs about knowing each other*. The themes and subthemes are listed in Table 5 in order of frequency.

Eleven of the 12 managers spoke of their own *beliefs about self-awareness* resulting in 31 references about such beliefs. Notions of the importance of being self-aware centered around the managers believing they must know what they do not know in order to learn. Five subthemes were identified including *the belief about seeking learning opportunities*, *the belief that one must be self-aware*, *the belief about own personal strengths and weaknesses*, *the belief about learning from employees*, and *the belief that one can only change oneself*. This theme is depicted through the following sample of quotes:

- Noel addressed the belief about seeking learning opportunities stating, “So, I seek out learning opportunities, and I have valued that. But the beauty of this stage of my life is that I recognize that opportunity is present in every interaction that I have…you know there’s probably a learning opportunity in any situation, good or bad, if you are open to what it’s trying to tell you.”
- Jim said of the belief that one must be self-aware, “…one of the things I’ve learned is, you cannot develop or grow if you are not aware of the areas you can develop in…we cannot change your habit if you are not aware of it to begin with…I mean if…I’m trying to think of an example. If somebody thinks they are a good listener, but all they do is talk, I do not know how you can fix that until they are willing to admit it.”
- Philip alluded to managers not knowing their vulnerabilities without being self-aware to “manage people who are doing work that you cannot yourself do, but still be able to contribute to it in such a way that it moves the work forward.”

The 11 managers who discussed the importance of being self-aware linked it to not only a knowledge of oneself, but the understanding that they might overcome their weaknesses through learning and utilization of employees’ strengths.

Eleven of the 12 managers spoke of their own *beliefs about learning* resulting in 26 references about the belief. Five belief subthemes were identified including *the belief that learning continually happens*, *the belief that all should and can learn*, *the belief that uncomfortable experiences lead to learning*, *the belief that one must seek to understand in order to learn*, and *the belief that learning is fun*. This theme is illustrated through the following sample of quotes:

- Noel stated, “The learning process is continually happening.”
- Brooke alluded to the belief that all should and can learn by stating, “Every day, you are going to learn. I think that’s it…just always be open to learning…and in this business, you better like learning ‘cause it changes…every client is different, every circumstance is different, every house is designed different, every piece of land is different…”
- Ken said, “I personally believe people hit developmental walls where they cannot move forward because they are paralyzed in some way…I do not have any other means to move, to be any better without some disruption.”

Nine of the 12 managers spoke of their own *beliefs about their roles as managers* resulting in 23 references about the belief. Five belief subthemes were identified *including the belief that managers should develop employees, the belief that the manager role is employee-role alignment*, *the belief that the manager role is employee learning and success*, *the belief that managers are to hold employees accountable*, and *the belief that managing comes naturally to the manager*. This theme is demonstrated through the following sample of quotes:

Many of the quotes from the managers revolved around the belief that managers’ roles include developing employees in role for the employees’ growth and that of the organization as illustrated by

- Randy spoke of the manager’s role saying, “The overall goal is to help them develop professionally within their role and hopefully, if they are in the right role, that professional development or progression helps them become fulfilled in their professional life…it’s just one part of their personal fulfillment.”
- Noel spoke of employee-role alignment stating, “It’s important to me to let people know, I’m not trying to change who you are, I’m trying to enhance so you get the outcomes you need in your role…make sure you are aligned with your best role.”
- Philip described employee learning and success within the manager’s role as goals saying, “My chief goal as a manager is their [employees’] success.”
- Ken added, “…the value I add for our company is in making sure and helping leaders like [employee name] be successful in their roles because if they are successful then our company is successful and so, it is, on one level, distilling that to a…in my mind…a key kind of coaching and support role to make sure he is equipped with everything that he needs, that he is supported the way he needs to be supported and that when he needs an ear or an encouragement or sometimes that can be a carrot and sometimes it can be a little bit of a challenge to put to him to light a fire, but that it is done in a manner that he ultimately embraces and is successful and therefore our company is successful.”

Eight of the 12 managers spoke of their own *beliefs about the context for facilitating learning* resulting in 20 references about the belief. Five subthemes were identified including *the belief that trust and honesty are important, the belief that psychological safety is important*, *the belief to be intentional about one-on-ones, the belief of a culture of respect*, and *the belief in systems* as depicted through the following sample of quotes:

- Charles articulated his and his organizational culture of facilitation of learning began where “[A]ny employee can walk in my office and talk to me, generally speaking, and that is true of everybody that is in management or supervision. So, we have an atmosphere of openness. We work very hard at being transparent and obviously, we cannot share everything with everybody, but what information that can be shared, we do share. “
- Noel alluded to the belief that psychological safety is important saying “We [the organization] want to create that environment where you can say, “I do not think that’s right,” and that be okay.”
- Noel said, “I’m always intentional about having one-on-ones” with employees.
- Brooke summarized, “I would say that in 15 years of my management, if I had to tell any manager one single thing that is the most important thing they do…is their one-on-ones with their teams and that is undivided attention.”

Eight of the 12 managers spoke of their own *beliefs about knowing their employees* resulting in 18 references about the belief. Five subthemes were identified including *the belief that individuals are different*, *the belief about employee strengths*, *the belief about relying on employee skills*, *the belief that people do not want to disappoint*, and *the belief that employee body language is telling*. This theme is illustrated through the following sample of quotes:

- Anna stated, “…her strength is working in the black and white. Her strengths have always been the numbers, the recs, following the rules, following the policies. Mine have not been.”
- Noel suggested, “We all have different personalities that serve or help us and sometimes they hinder us, but they are always going to be a part of who we are right?”
- Ken said, “Everybody has a unique set of talents and abilities, and some of that kind of goes under the umbrella of, you know, we are all just wired a little differently.”

Eight of the 12 managers spoke of their own *beliefs about how to manage more developmentally* resulting in 16 references about the belief. The beliefs about how to manage differ from the beliefs about the role as manager because they address more of the style, the process, or the approach and not just the what, or the goals of managing. Eight subthemes were identified including *the belief that authoritative management is bad*, *the belief about managing with flexibility*, *the belief that managers lead by example*, *the belief that everyone is a leader*, *the belief that communication is important*, *the belief that one should keep a big-picture perspective*, *the belief that listening is important*, and *the belief that managing is like parenting* as depicted through the following sample of quotes:

- Charles said, “Even though I have the authority to tell anybody what they should do, if I exercise that, that is not being a very good boss…If I do not think I am getting where I have to go, and I have to play the card, no you are going to do this, then I really have not coached now. I am just ordering people around; and now, I have become a supervisor dictator, and they do not learn anything from that.
- Ken spoke of leading by example stating, “I personally subscribe very strongly to a theory that much learning comes through imitation and modeling behavior that employees see…I do not think it’s fair for a manager to ask someone to do something that they are unwilling or unable to do themselves.”
- Noel, when discussing how to know how to manage, stated, “I think it’s through the conversation of just making sure you stay with that conversation long enough to close the loop.”
- Noel addressed another belief that one should keep a big-picture perspective, “We do not want to get pennywise and pound foolish is a term where I am thinking about the policy and not thinking about what the policy is designed to do.”
- Scott illustrated the belief that listening is important by stating, “I just think it’s something where you get where you listen. I’m not saying it all applies, but if you’ll listen to people.”

Five of the 12 managers spoke of their own *beliefs about knowing each other* resulting in 10 references about the belief. Three subthemes were identified including *the belief that informal relationships are important, the belief that the personal and professional affect each other*, and *the belief that assessments have a purpose* as illustrated through the following sample of quotes:

- Ken stated, “One of the things that I think is really critical to a manager’s development and, well, and any employee’s development, is cultivating informal partnerships and informal relationships within the organization.”
- Charles addressed work assessments being tools to better understand each other mentioning, “Assessments do not tell you what somebody is, but they tell you the questions to ask…they give you a direction to go in, but we are much more complicated than a score.”

Three additional themes with six subthemes emerged from the *Employees’ Perspectives as Coachees of Managers’ Beliefs When Serving as Coaches*. The broad themes include *beliefs about employee*, *beliefs about management style*, and *belief about learning*. The themes and subthemes are listed in Table 5 in order of frequency.

Five of the 12 employees spoke of *their managers’ beliefs about them, the employees*, resulting in 6 references about the belief. Two subthemes were identified including *the belief in employee capabilities*, and *the belief about trusting the employee*. This theme is depicted through the following sample of quotes:

- Natalie, in speaking about the beliefs they thought their managers held about them, said, “I think she knows sometimes what me and [colleague] and all of her direct reports are capable of before we do sometimes.”
- Daisy mentioned, “She [manager] has the same bar for everybody…it’s high…but, it’s the same bar…she wants you to bring your full potential.”
- Penny illustrated the managers’ belief about trusting the employee by simply stating, “…I feel like he [manager] trusts me.”

Three of the 12 employees spoke of *their managers’ beliefs about management style* resulting in 4 references about the belief. Three subthemes were identified including t*he belief that the manager values relationships*, *the belief that the manager has positive intent*, and *the belief that the manager values communication*. This theme is demonstrated through the following sample of quotes:

- Daisy illustrated the belief that the manager has positive intent by stating, “I know that her [manager’s] feedback is coming from a good place because she knows who I am.”
- Allison perceived the that the manager believes in valuing relationships by saying, “…that [her manager] is relationship driven. And when you remember that relationships aren’t just about developing relationship to sell, developing relationships from a client, from a donor…it’s not just about the external people it’s the team that you work with and really being open and caring and finding out more about those people that you work with…just makes you more open and kinder and gentler.

Two of the 12 employees spoke about *their managers’ beliefs about learning* resulting in 2 references about the belief. This theme is illustrated through the following sample of quotes:

- Allison acknowledged, “I think it’s important for her to be the best CEO she can be.”
- Brenda declared, “They’re [manager are] real open to change…”

## 4. Discussion

The findings associated with managerial coaching behaviors reported in this study offer support and reinforce the behaviors included in several previously developed behavioral taxonomies. Specifically: provides feedback (Ellinger, 1997; Ellinger et al., 1999; Beattie, 2002; Ellinger et al., 2003; Ellinger, 2005; Heslin et al., 2006; David and Matu, 2013), providing accountability (Amy, 2008), prioritizing and organizing (Beattie, 2002; Amy, 2008; David and Matu, 2013), delegating (Beattie, 2002; Hamlin, 2004; Amy, 2008), intentionally scheduling meetings (Ellinger, 1997; Ellinger et al., 1999; Ellinger, 2005; Heslin et al., 2006; David and Matu, 2013), listening (Hamlin, 2004), leading by example (Beattie, 2002; Ellinger, 2005), empowering others (Beattie, 2002; Hamlin, 2004; Heslin et al., 2006; Amy, 2008; Gilley et al., 2010; David and Matu, 2013), promoting and developing employees (Beattie, 2002; Hamlin, 2004; Ellinger, 2005; Amy, 2008; Park et al., 2008; Gilley et al., 2010; Gregory and Levy, 2010), adjusting style for individual employees (Amy, 2008), and establishing rapport through trust and communication (Ellinger et al., 1999; Beattie, 2002; Hamlin, 2004; Amy, 2008; Park et al., 2008; Gilley et al., 2010; Gregory and Levy, 2010).

Most behavioral taxonomies that have been created are based upon managers’ perspectives about their self-reported enacted behaviors. However, the findings from this study sought to obtain employee perspectives about the behaviors they experienced when their managers coached them. The findings from this study relating to the perceptions of the employees as coachees corroborate many of the behavioral findings that their managers reported. Employees mentioned that the managers’ behaviors included developing employees (Beattie, 2002; Ellinger, 2005; Amy, 2008; Park et al., 2008; Gilley et al., 2010; Gregory and Levy, 2010), communicating expectations (Ellinger et al., 1999, 2003; Park et al., 2008; Gilley et al., 2010; Gregory and Levy, 2010), intentionally scheduling meetings (Ellinger, 2005; Heslin et al., 2006; David and Matu, 2013), leading by example (Beattie, 2002; Ellinger, 2005), providing feedback-correction (Ellinger et al., 2003; Ellinger, 2005; Heslin et al., 2006; David and Matu, 2013), exhibiting patience-forgiveness (Beattie, 2002), and prioritizing (Amy, 2008; David and Matu, 2013).

In terms of managers’ beliefs about coaching, the limited literature that has explored the beliefs or dispositions of managers has focused on those perceived by the managers (Ellinger and Bostrom, 2002; Misiukonis, 2011; Campbell and Evans, 2016). From the perspective of the managers, this study found that managers who coach held beliefs about self-awareness that align with Ellinger and Bostrom’s (2002) research on beliefs about managers serving as facilitators of learning, particularly the cluster of self-efficacy of the managers’ own strengths, as well Anderson’s (2013) occupational self-efficacy, and Campbell and Evans (2016) managers’ self-belief, and Misiukonis (2011) beliefs about self-efficacy. Managers’ beliefs about learning found in this study reinforce existing literature, but with differing subthemes or clusters. For example, the subtheme of learning being continual was found in this study and that of Ellinger and Bostrom (2002) but the subtheme that learning is fun emerged as new in the study.

Campbell and Evans (2016), Ellinger and Bostrom (2002), Ellinger et al. (2018), Hunt and Weintraub (2017), and Misiukonis (2011) also found managers’ beliefs to include those about the manager’s role relating to this study’s finding that managers should develop employees; beliefs about creating the right environment (Campbell and Evans, 2016); my role is to facilitate learning and development (Ellinger and Bostrom, 2002); a coaching mind-set consists of a desire to help others to develop (Hunt and Weintraub, 2002, 2017; Ellinger et al., 2018). One of the study’s subthemes of the theme of managers’ beliefs about context for facilitating learning, the belief that trust and honesty are important, relates to Ellinger and Bostrom’s (2002) cluster that the best learning occurs when caring and trusting relationship exist. The subthemes of beliefs about learning that all should and can learn as well as uncomfortable experiences lead to learning were not overtly found in the reviewed literature but emerged in this study. Campbell and Evans (2016) tangentially touch on a manager believing that learning is uncomfortable when finding that managers believe people need support to develop confidence.

The study’s theme and subthemes relating to managers’ beliefs about knowing their employees supports Campbell and Evans (2016) belief that people are individuals and Misiukonis (2011) beliefs about subordinates. The study’s beliefs about how to manage more developmentally, particularly the belief that managers lead by example, directly relates to Campbell and Evans (2016) managers’ belief that leaders are role models. Lastly, the belief theme reported here regarding managers’ beliefs about knowing each other, and subtheme that informal relationships are important, loosely aligns with Ellinger and Bostrom’s (2002) manager beliefs that the manager cares enough to help employees learn and that the best learning occurs when caring and trusting relationships exist.

The overall theme found in this study about managers’ beliefs about context for facilitating learning may be found in Campbell and Evans’ (2016) belief of creating the right environment and Ellinger and Bostrom’s (2002) belief that establishing trust and building relationships is critical. However, some of the subthemes in this study offer new insights regarding how the environment may be created or facilitated. For instance, the managers’ belief that psychological safety is important emerged as a new belief in this study.

Of the limited research that has explored managers’ beliefs about coaching, the beliefs articulated by managers have been self-reported, However, this study sought to better understand beliefs that managers hold from the perspective of the employees that the managers coach since they are the recipients of the managers’ intentions. The findings reported here from the perception of the employees as coachees corroborate many of the findings that are reported by managers. For example, the belief that managers value relationships aligns with Ellinger and Bostrom’s (2002) belief that the best learning occurs when caring and trusting relationships exist. Lastly, the theme, belief about learning, from the employees’ perspective aligns with both Campbell and Evans’ (2016), Ellinger and Bostrom’s (2002), and Hunt and Weintraub’s (2002, 2017) thematic categories of managers’ beliefs about learning and the learning process. A belief subtheme from the employees’ perspectives emerged that the manager has positive intent. This supports and reflects their managers’ beliefs about context for facilitating learning including the beliefs that trust and psychological safety are important. When employees intuit their managers’ beliefs about them, their relationships, and the learning process that unfold when their managers are coaching them, and there is resonance with the beliefs that their managers hold as coaches, the authenticity in the behavioral intentions is realized.

In summary, the qualitative data collected and analyzed in this study reinforces and supports much of the existing research on managerial coaching behavior and behaviors reported in a number of behavioral taxonomies that have been created. However, it specifically extends the literature by examining the perspectives of the employees being coached which corroborates much of the self-reported behaviors by managers. A new and important finding from this study is the theme that emerged about managers’ self-reported behavior of commitment to self-learning that seemed to be perceived by their employees as coachees as role-modeling self-induced learning. This specific behavior of managers, under-represented in the current literature, suggests that such self-learning builds coaching capacity and enhances the coaching behaviors that managers enact thus ensuring that what managers espouse is exhibited in their subsequent behaviors. In contrast to behaviors, managerial beliefs about coaching have not been extensively explored. The findings reported here do offer support for many of the overarching belief categories that have been previously identified in the few studies that have articulated such beliefs. However, there are several new insights that have been articulated in many of the subthemes that emerged. In addition to the self-reported beliefs of managers which further add to the limited existing research, this study also incorporated employees’ perceptions of their managers’ beliefs about coaching, thus offering corroboration of specific beliefs relating to the importance of relationships and the learning process.

## 5. Contributions to Research, Theory, and Implications for Practice

This section articulates the contributions that this study makes to research, theory, and offers specific implications for practice. It also discussion limitations and future research directions.

### 5.1. Contributions to research and theory

The behavioral findings reported here offer support for and reinforce much of the existing literature that has previously examined managerial coaching behaviors and resulting taxonomies. Our findings also further extend the managerial coaching behavior literature by offering some corroboration from coachees who have often not been included in such studies of managerial coaching behavior.

In terms of beliefs, scholars have called for more research that explores managers’ beliefs about coaching. This study addresses these calls by obtaining discrete critical incidents of managers’ self-reported behaviors when they coached their employees and then specifically sought to better understand managers’ beliefs that inspired and encouraged them to coach and guided their behaviors from both the perspectives of managers who coach and their respective employees. Soliciting employees’ perceptions of their managers’ beliefs helped to corroborate some of these beliefs. Furthermore, while the findings on beliefs reported here resonate with many of the overarching beliefs categories derived in the few other studies that have explored managerial coaching beliefs, the findings reported here offer some new insights within these broader categories of beliefs through the articulation of the subthemes and rich illustrations that have been provided.

**Figure 1:**
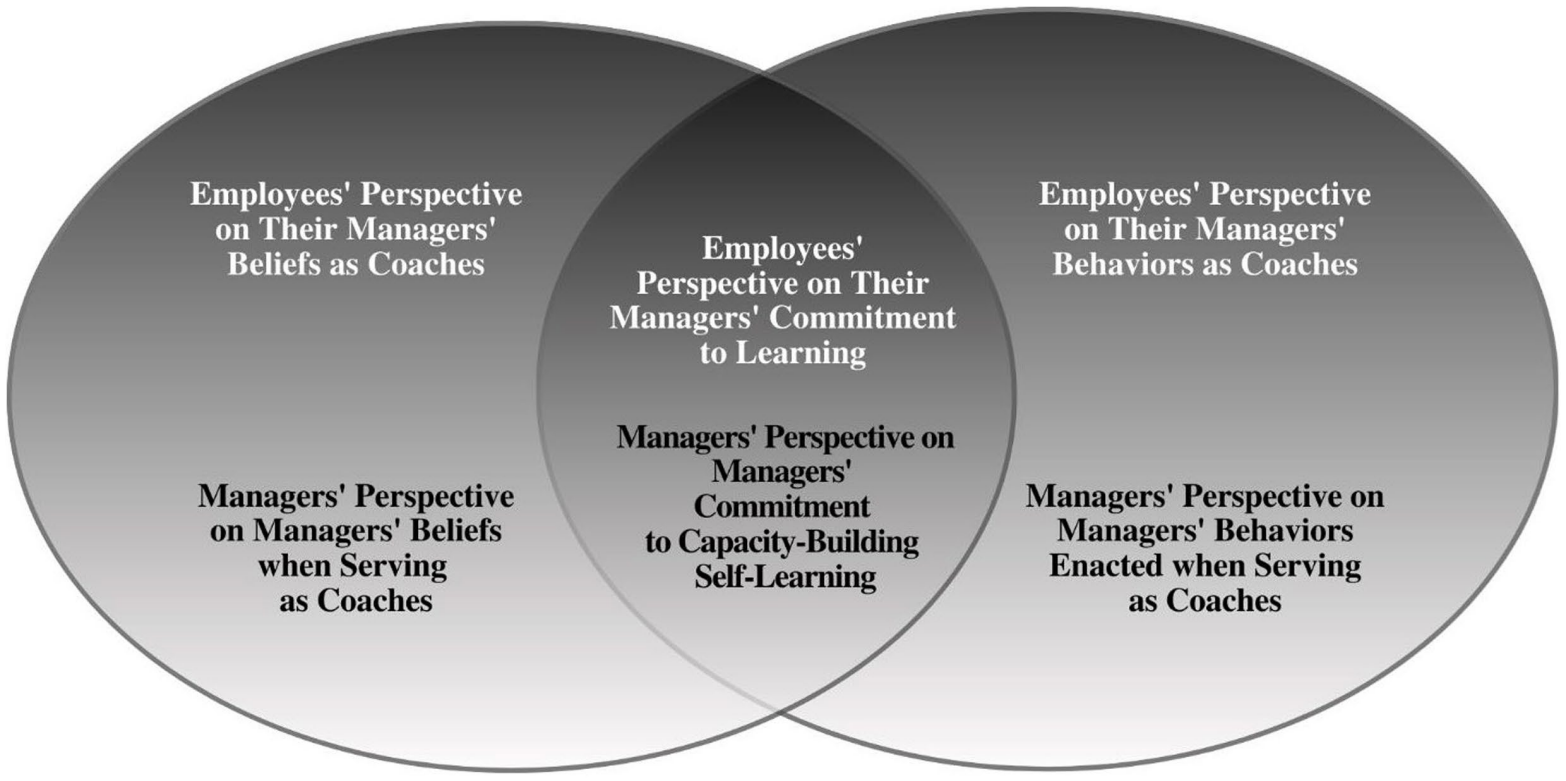
Managers’ and employees’ perceptions of managers’ coaching beliefs and behaviors.

Furthermore, a specific new managerial behavior that seemingly influences their coaching reported in this study suggests that managers who are committed to self-learning through their approaches to engage in continual learning, not only promotes building coaching capacity for managers which can further enhance their behaviors, but models the importance of self-induced learning to their employee coachees. This behavioral theme, depicted in Figure 1, may indeed represent an important mechanism that may serve as a linkage between coaching beliefs and enacted behaviors by affecting the efficacy and subsequently the outcomes of the managerial coaching intervention. From this study, it is clear that managers’ beliefs about seeking learning opportunities and beliefs that all can and should learn appear to manifest in their own commitment to self-learning.

In terms of theory, the enacted behaviors and the beliefs articulated by the managers that guide these behaviors are consistent and does offer a contrasting perspective to the contradictions that Argyris and Schön (1996) and Argyris (1991) acknowledge often exist between the managers’ espoused theory and their theory-in-use. We suggest that this consistency may be attributed to managers’ commitment to learning and the possibilities that their beliefs may have evolved over time as they became exemplary coaches.

### 5.2. Implications for practice

There are a number of implications from this study that can inform managerial coaching practice. The findings reported here about managerial coaching behaviors offer further support for and reinforce much of the existing literature on such behaviors, and the content of many behavioral taxonomies that guide managerial coach training. Thus, our findings offer additional credibility for advocating for such behaviors, and for inclusion in such coach training programs developed for managers, and can be used for observing such behaviors, and for surveying such behaviors. With regard to managerial coaching beliefs, the findings reported here also provide more insight into the antecedents for managerial coaching behavior, from exemplary managers who serve as coaches, along with impressions of beliefs from those employees who are coached. These antecedents could influence the development of a taxonomy of observable, thus assessable, beliefs of managers who have the propensity for engaging in coaching and enacting coaching behaviors that can enable their employees to grow, develop and improve their performance. Such articulated beliefs can be used by managers as a form of self-reflection about what their roles and responsibilities are, could, or should be with regard to serving as managerial coaches. Furthermore, a managerial coaching beliefs scale could be developed and deployed which would enable researchers and practitioners to further determine which beliefs most favorably influence specific coaching behaviors and coaching outcomes. At present, research linking specific coaching behaviors with specific coaching outcomes is considerably under-developed as is research connecting coaching beliefs with coaching behaviors given the limited belief research that has been undertaken. The behavioral and belief findings reported here may also be informative to human resource development and management professionals who are responsible for crafting job descriptions, job qualifications, interview questions, and hiring assessments, along with the provision of training and organization development interventions to encourage managerial coaching and promote the development of a coaching culture. Based upon the findings reported in this study and in relation to previous literature on managerial coaching behaviors and beliefs, we offer a summary table that highlights what overarching managerial coaching beliefs and behaviors are consistently held in common to further guide managerial coaching practice (see Table 6).

**Table 6:** Summary of most commonly held beliefs and behaviors of managers who coach.

Beliefs	Behaviors
Beliefs about self: Beliefs about self-awareness Beliefs about self-efficacy Beliefs about the manager’s role: Beliefs about developing employees Beliefs about creating a positive learning environment Beliefs about facilitating learning Beliefs about trust and honesty being important Beliefs about learning occurring when a trusting relationship exists	Asks questions Provides feedback Sets expectations and standards Provides accountability Effectively communicates with employees Delegates to employees Listens to employees Empowers employees Develops employees

### 5.3. Limitations and future research recommendations

A number of limitations should be acknowledged here with regard to the sampling procedure, sample size, and data collection techniques that were employed in this larger study. For example the subjective opinions of nominators and the resulting pool of exemplary managers may have influenced the findings, although the criteria for selection was fairly rigorous regarding whom nominators deemed as exemplary managerial coaches. Since managers also nominated their respective employee, it is possible that some bias may have been possible that could influence the findings. However, given the corroboration that occurred during separate interviews, this may be less likely. A total of 24 participants comprising 12 cases, or managerial coaching dyads, was sufficient for the purposes of this research. Yet, it should be noted that this purposeful selection included eight organizations for which these dyads were embedded which is not representative of all industries where managerial coaching may be practiced. This study was also delimited by a specific geographical location of the south-central United States thus requiring consumers of this research to consider geographical limitations. In terms of beliefs, we acknowledge our assumption that beliefs guide behaviors, but recognize that other scholars may hold differing perspectives and assumptions. It is also possible that, since beliefs were captured following the articulation of their critical incidents which reflected their self-reported behaviors, managers’ beliefs may have evolved over time. We sought to obtain what managers’ think they do (their enacted behaviors) when they coach, and then why they do (their articulated beliefs) what they do when they coach. However, given the nature of our design, we are unable to establish causal links to determine which specific beliefs guide which specific behaviors. This could be a fruitful line of future research, particularly if a beliefs scale was developed that could be used in relationship with existing managerial coaching behavioral scales. Given some of these limitations, more contextual, social and cultural nuances, and industry sector influences could be examined beyond the scope of this study. Additional future research studies could provide a richer understanding about the intersectionality of race, gender, ethnicity, and culture on the composition of the coaching dyad and the coaching relationship as it relates to beliefs. From a design perspective, conducting separate interviews, and then interviewing the dyad members together at various points during data collection may yield additional insights about shared critical incidents along with aspects of the managerial coaching dyad relationship which has been underexplored (DeHaan and Gannon, 2017). Although beliefs take time to form and may evolve and change over time, it might be insightful to understand how managers’ beliefs may change throughout their coaching practice by examining a range of managers who coach in terms of their ages and experience levels.

## Data availability statement

The original contributions presented in the study are included in the article/supplementary material, further inquiries can be directed to the corresponding author.

## Ethics statement

The studies involving human participants were reviewed and approved by The University of Texas at Tyler Institutional Research Board. The patients/participants provided their written informed consent to participate in this study. Written informed consent was obtained from the individual(s) for the publication of any potentially identifiable images or data included in this article.

## Author contributions

BA and AE contributed to the conception and design of the study, engaged in the writing and editing of this manuscript and approve of the submitted manuscript and supplemental material. BA collected and analyzed the data. AE reviewed the data and contributed to the analysis process. All authors contributed to the article and approved the submitted version.
